# Melanin-based structural coloration of birds and its biomimetic applications

**DOI:** 10.1186/s42649-021-00063-w

**Published:** 2021-10-11

**Authors:** Deok-Jin Jeon, Suejeong Paik, Seungmuk Ji, Jong-Souk Yeo

**Affiliations:** 1 School of Integrated Technology, Yonsei University, 85 Songdogwahak-ro, Yeonsu-gu Incheon, 21983 Republic of Korea; 2 Yonsei Institute of Convergence Technology, Yonsei University, 85 Songdogwahak-ro, Yeonsu-gu Incheon, 21983 Republic of Korea; 3 39 Yeonhui-ro 22-gil, Seodaemun-gu Seoul, 03723 Republic of Korea

**Keywords:** Melanin, Structural color, Pigment, Biomimetics

## Abstract

Melanin has been a widely researched pigment by scientists for decades as it is undoubtedly the most ubiquitous and ancient pigment found in nature. Melanin plays very significant roles in structural plumage colors in birds: it has visible light-absorbing capabilities, and nanoscale structures can be formed by self-assembling melanin granules. Herein, we review recent progress on melanin-based structural coloration research. We hope that this review will provide current understanding of melanin’s structural and optical properties, natural coloration mechanisms, and biomimetic methods to implement artificial melanin-based structural colors.

## Introduction

Most animals survive by obtaining information from colors. Colors can be used for communication between conspecifics, and colors can also be used to convey subordination signals, nutritional conditions, health quality, and even genetic conditions. Particularly, birds are representative animals that display vivid, bright, and colorful colors. Over 10,000 species of birds possess and utilize an amazing diversity of colors in their feathers (Brusatte et al. 2015; Hart and Vorobyev 2005; Jetz et al. 2012; Lovette 2014; Hill and McGraw 2006; Tedore and Nilsson 2019). As birds are tetrachromats, birds are more sensitive to a wider range of color spectrum in comparison to humans. Humans have three types of cones or photoreceptor cells (peaks at 424, 530, and 560 nm). In contrast, birds have four types of cones (peaks at 370, 445, 508, and 565 nm). Because of these advantages, research on birds’ plumage colors has attracted increasing scientific attention.

In birds’ plumage, pigments and structures are two main sources that generate color (Auber 1957; Hill and McGraw 2006). First, pigments make color by using chemical molecules that absorb specific wavelengths. Second, unlike pigment-based colors, structure makes color by selectively reflecting light of certain wavelengths from nanostructures. Melanin is a type of pigment that contributes to the coloration of both pigment-based and structural colors of feathers (Hill and McGraw 2006; Riedler et al. 2014). In fact, melanin itself can function as a pigment that absorbs visible light and simultaneously produce structural color. Overall, melanin plays a significant role in the production of bird coloration, thus serving as a major component.

Melanin has been a widely studied pigment by scientists for decades because it is undoubtedly the most ubiquitous and ancient pigment found in nature (d'Ischia et al. 2014; d'Ischia et al. 2020; Simon and Peles 2010). Traditional and more recent technologies, such as scanning electron microscopy (SEM), transmission electron microscopy (TEM), atomic force microscopy (AFM), electron energy loss spectroscopy (EELS), and energy-dispersive spectroscopy (EDS) have enabled huge advances in understanding the molecular structures, optical properties, and coloration mechanisms in the last few decades (Galeb et al. 2021). Over the last decade, several papers have reviewed melanin chemistry, optical functions of melanin, melanosome morphology, and biomimetic structural coloration (D'Alba and Shawkey 2019; Kohri 2019, 2020; Sun et al. 2013; Xiao et al. 2020). However, there is a lack of a comprehensive review focusing on how optical and structural properties of melanin are related to biomimetic applications. In this review, we cover current issues of melanin-based colors of birds and briefly summarize biomimetic optical applications inspired by natural melanin-based colors.

## Physical mechanisms of structural colors in birds

Structural colors in birds are generally observed on barbs or barbules of feathers. β-keratin nanostructures and melanin are the major factors affecting colors in barb-based structural colors, whereas β-keratin cortex and melanin are the major factors in barbule-based structural colors. β-keratin nanostructures and cortex are factors that only affect the reflection of certain wavelengths, whereas melanin affects the reflection and absorption. Simultaneously, melanin plays a role in not only absorbers of broadband UV to visible light but also building blocks for photonic nanostructures. Structural colors are generated by complex interactions between incident light and optical components, especially melanin and β-keratin in feathers. Structural colors in birds can be classified into five categories: thin-film interference, multi-film interference, coherent scattering, incoherent scattering, photonic band gap formation by photonic nanostructures.

**Fig. 1:**
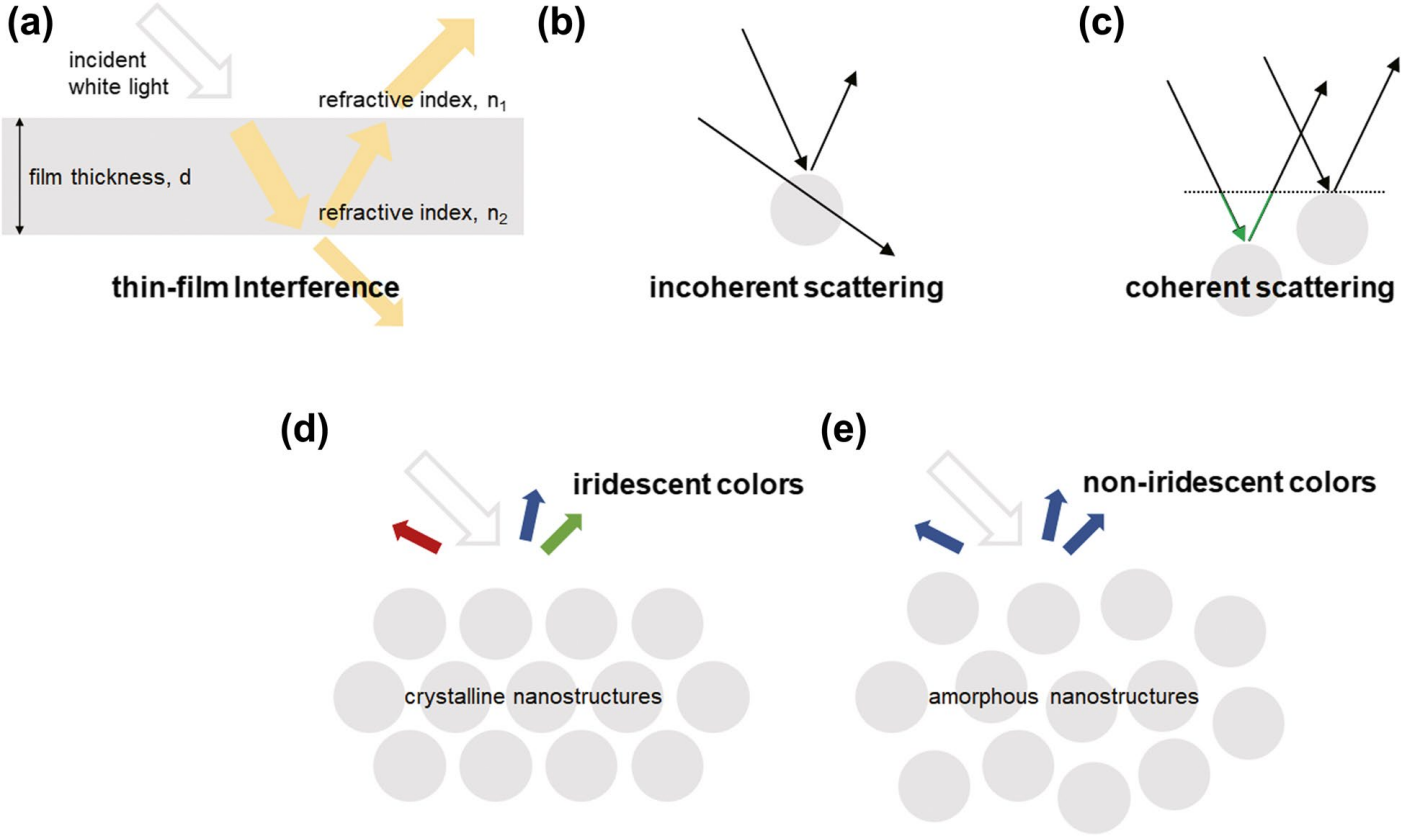
(**a**-**c**) Basic physical mechanisms of structural coloration in birds. Structural colors appeared in barbs or barbules are based on (**a**) Thin-film interference, (**b**) Incoherent scattering, (**c**) Coherent scattering, (**d**-**e**) Representative melanin-based photonic nanostructures, (**d**) Crystalline nanostructures generate iridescent colors, and (**e**) Amorphous nanostructures generate non-iridescent colors

Thin-film interference is a phenomenon frequently observed in nature. A simple example of structural colors caused by thin-film interference is a rainbow iridescent colors of a thin-film of oil on water. As shown in Fig. 1(a), a thin-film material surrounded by other materials consisting of different refractive indices can cause thin-film interference. Light reflected from both top and bottom interfaces of a thin-film can be cancelled out or reinforced (Pancharatnam 1956; Parker 2000). Reflected light waves that are out of phase will destructively interfere, whereas reflected light waves that are in phase will constructively interfere. Peak wavelength of structural colors caused by thin-film interference can be defined as

$$\lambda =\frac{2{n}_2 dcos\theta}{m}$$

where *λ* is the peak wavelength, *n*_2_ is a refractive index of thin-film material, *d* is a film thickness, *θ* is an angle of refraction, and *m* is an arbitrary natural number. Multi-film interference can be simply understood in terms of thin-film interference with multilayer arrangement. In birds, structural colors from thin-film interference are observed in the barbule-based structural colors. β-keratin cortex of barbule can act as a thin-film material. Multilayered thin-film structures make light waves reflect multiple times at the interfaces. Structural colors from multi-film interference often appear to be highly saturated, vivid, and bright when compared to thin-film interference (Sun et al. 2013). Birds’ multilayered melanosomes can produce structural colors from multi-film interference.

Light scattering means light-matter interactions with scattering objects such as elementary particles (Skipetrov and Sokolov 2014). In conventional use, scattering includes constructive or destructive interferences of light waves reflected by light scattering objects (Prum et al. 2006). In addition, light scattering for structural colors in birds can be simply classified into two categories: incoherent scattering and coherent scattering. Incoherent and coherent scatterings differ in the dependency of the phase relationships (Prum and Torres 2003). In incoherent scattering, there is no phase relationship of scattered light waves as shown in Fig. 1(b). In contrast, there is a phase relationship of scattered light waves in coherent scattering, because coherent scattering occurs when scattering objects have uniform arrangement as shown in Fig. 1(c). In birds, β-keratin nanostructures and melanosomes can act as scattering objects.

Optical properties of structural colors in birds are also dependent on the crystallinity of photonic nanostructures. As shown in Fig. 1(d, e), crystalline photonic nanostructures (photonic crystals, PhCs) that have a long-range order produce angle-dependent iridescent colors, whereas amorphous photonic nanostructures (photonic glasses, PhGs) that have a lack of long-range order produce angle-independent non-iridescent colors. There is the trade-off relationship between color saturation and angle-dependency. Therefore, compared to PhCs, PhGs display less saturated structural colors. Traditionally, it was known that only long-range ordered PhCs can form photonic band gap (PBG), but the recent study support that short-range ordered PhGs can also form PBG (Miyazaki et al. 2003). In birds, natural photonic nanostructures are composed of β-keratin nanostructures in barbs or melanosomes in barbules. Particularly, in PhGs-type melanosomes, melanin’s strong absorption properties help to achieve saturated and bright structural colors by suppressing incoherent scattering of light.

## Structural and optical properties of melanin

Melanin is a pigment most commonly produced by melanocytes within intracellular melanin granules (melanosomes) (Simon and Peles 2010). Melanin has an aggregation of monomer building blocks that form biopolymers (King 2007). Melanin is produced by various organisms including bacteria, fungi, plants, animals, birds, and humans (Cordero and Casadevall 2020; D'Alba and Shawkey 2019). Structural and optical properties of melanin are highly dependent on chemical and hierarchical structure of melanin (D'Alba and Shawkey 2019). Therefore, the understanding on melanin chemistry, melanosome morphology, and organization is highly demanded.

There are four general types of melanin: eumelanin, pheomelanin, neuromelanin, and allomelanin (Nicolaus 1968; G. Prota 1988; Singh et al. 2013). Eumelanin and pheomelanin are most commonly found in birds and other animals including humans (D'Alba and Shawkey 2019). Neuromelanin is a dark pigment found in the brain. Neuromelanin assists navigation with electromagnetic tracking (Bolzoni et al. 2002; Sakurai et al. 2004), and reduces oxidative stress in the brain (Xiao et al. 2020). Allomelanin is a heterogeneous group of nitrogen-free polymers found in bacteria, fungi, and plants (Nosanchuk and Casadevall 2003). This review focuses on eumelanin and pheomelanin because these are the two main pigments in birds’ plumage coloration.

**Fig. 2:**
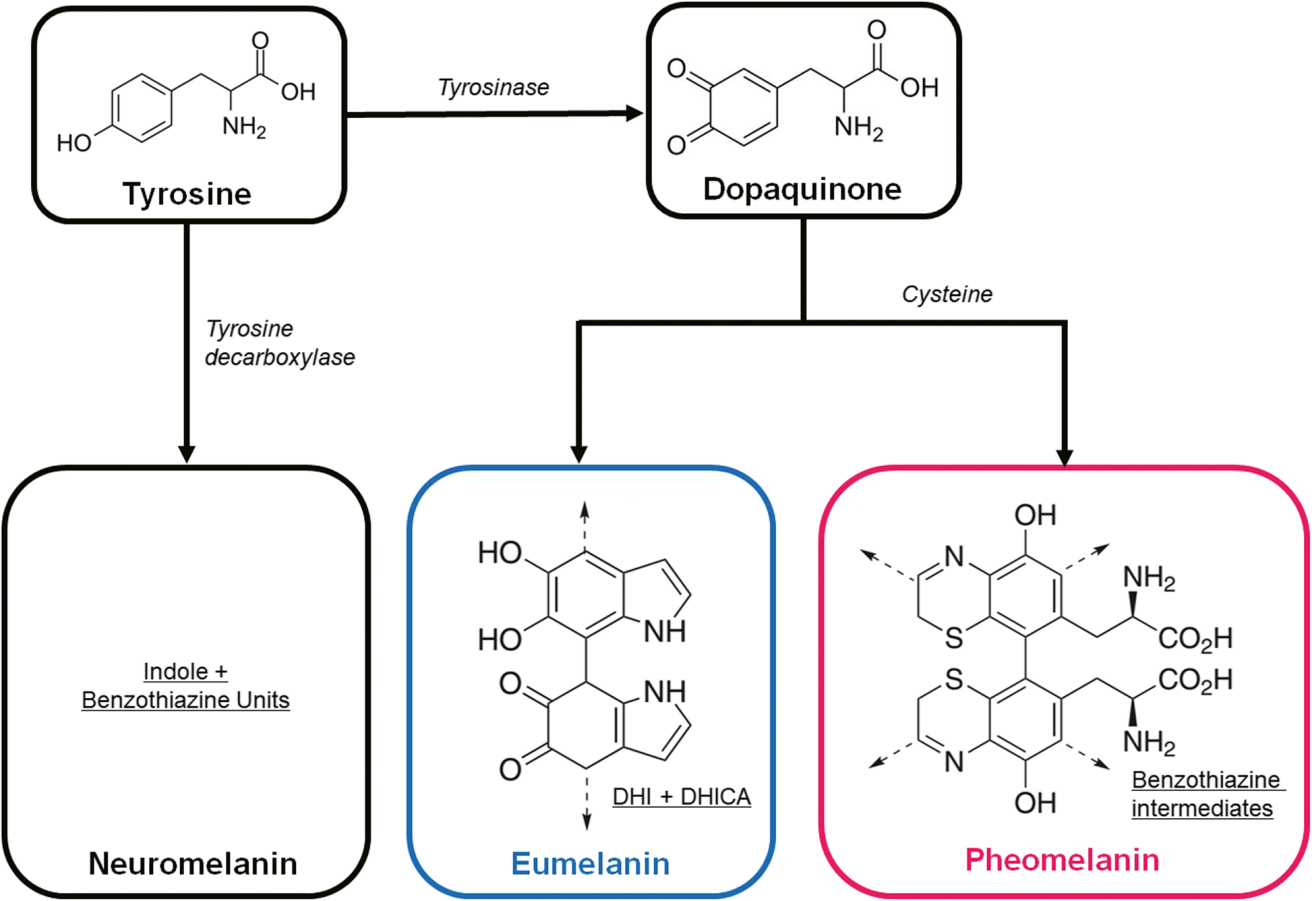
Biosynthetic pathways of three different types of natural melanin in birds

In biosynthetic systems, types of melanin are produced by following oxidative processes that involve enzymes such as oxidases. For example, eumelanin and pheomelanin are produced within melanocytes by the biosynthetic pathway that includes the tyrosinase-involved oxidation of tyrosine (Fig. 2). Eumelanin and pheomelanin could be conventionally classified based on differences in their optical properties: eumelanin shows black to brown while pheomelanin shows yellow to reddish colors (Prota 1992; Simon and Peles 2010). Recent studies found that eumelanin is composed of two molecules: 5, 6-dihydroxyl indole (DHI) and 5, 6-dihydroxyindole-2-carboxylic acid (DHICA) (Miserez et al. 2008). Based on advances in X-ray and high-resolution TEM techniques, these two eumelanin protomolecules are shown in planar structures by π-π stacking (Cheng et al. 1994; d'Ischia et al. 2013; Watt et al. 2009; Xiao et al. 2018). Pheomelanin is assumed to be composed of benzothiazine intermediates (Simon et al. 2009). There is, however, still inadequate understanding of pheomelanin’s chemical structure, therefore requiring further studies on melanin oligomers’ chemical structures and synthetic processes based on intramolecular interactions. Additionally, in birds and mammals, one significant problem in research on melanin is that eumelanin and pheomelanin are often observed in a combined form. Most melanin-based colors are generated from a combination of eumelanin and pheomelanin in varying concentrations. Quite a few of papers have reported melanosomes containing pure eumelanin or pheomelanin (Ito and Wakamatsu 2003; Hill and McGraw 2006; Micillo et al. 2017; Naysmith et al. 2004; Simon et al. 2008). The difficulty separating types of melanin makes it difficult to interpret optical functions of melanin-based colors.

**Fig. 3:**
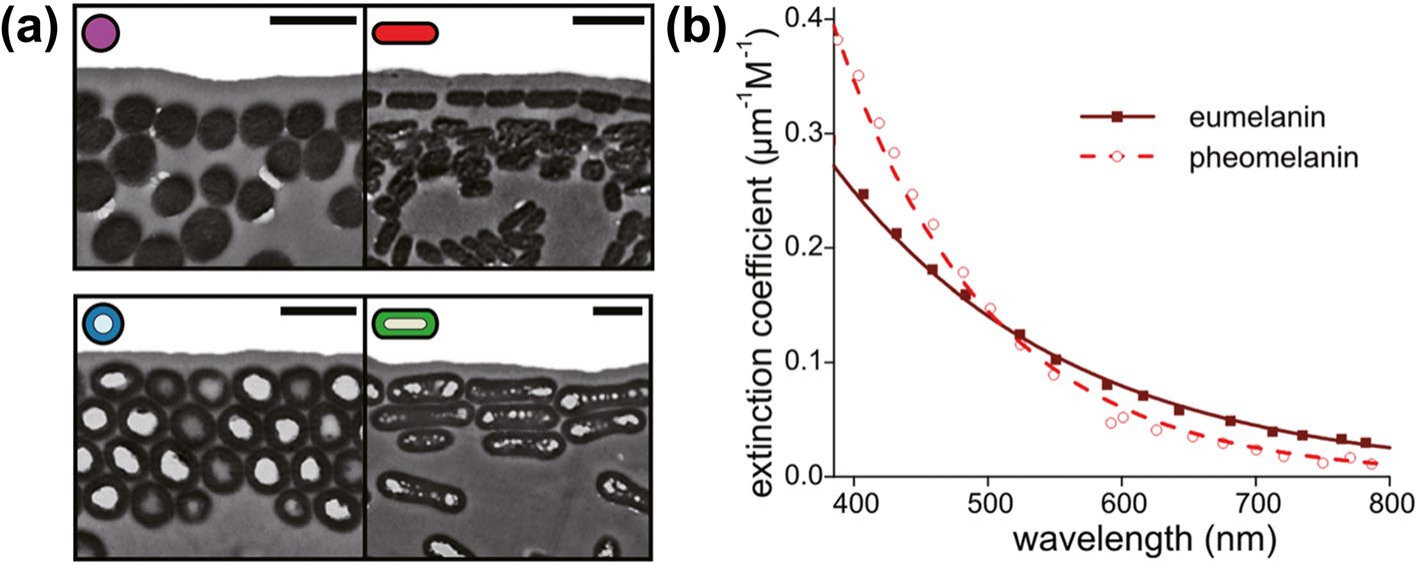
Structural and optical properties of melanin. (**a**) Melanosomes in birds have four main structures: nanoparticles (purple), nanorods (red), hollow nanorods (blue), and hollow platelets (green). (Scale bars: 500 nm). Reproduced with permission (Maia et al. 2013). Copyright 2013, The Authors. Published by National Academy of Sciences. (**b**) Extinction coefficients of eumelanin and pheomelanin in the visible range. Reproduced under the terms of the Creative Commons CC BY license (Stavenga et al. 2012). Copyright 2012, The Authors. Published by the Public Library of Science

Melanosomes can be in spherical, rod, hollow-rod, or hollow-platelet shapes as shown in Fig. 3(a). Based on previous findings about melanin coloration, larger percentages of eumelanin can form spherical, rod, hollow-rod, and hollow-platelet melanosomes, but nearly pure pheomelanin can only form spherical-shaped melanosomes (Durrer 1986; Liu et al. 2005; Maia et al. 2013). Various melanosome morphologies may be associated with the chemical structure of melanin, and there are many efforts to investigate melanin and melanosomes. However, scientists encounter significant technical limitations to accurately characterize individual melanosomes. More research on investigating the chemistry of melanin and melanosomes may further clarify the correlation between the chemical structure of melanin and melanosome morphologies.

Melanin has a unique and distinguishing feature on the broadband absorbance spectrum as shown in Fig. 3(b): eumelanin and pheomelanin absorb visible light, eumelanin absorbs more than pheomelanin for longer wavelengths (> 500 nm), and eumelanin and pheomelanin absorption spectra do not have peaks (Stavenga et al. 2012). While most biological pigments show distinct peaks, this monotonic absorption spectrum differs from other biological pigments (Xiao et al. 2020). The monotonic absorption of melanin differs also from broadband absorbers such as carbon black. Melanin absorbs more ultraviolet (UV) and blue light than longer wavelengths. There are several studies that have contributed to the cause of this monotonic absorption of melanin (Chen et al. 2014; McGinness et al. 1974; McGinness 1972; Meredith et al. 2006; Tran et al. 2006). For instance, Meredith et al. suggested that the large broadband absorption spectrum was caused by the superposition of absorption of all spectra at different wavelengths (Meredith et al. 2006). Chen et al. found that the interplay of geometric order and disorder of eumelanin aggregate structures could broaden the absorption spectrum (Chen et al. 2014). The complexity of melanin structure is the main cause of our understanding on the monotonic absorption properties of melanin.

## Melanin-based structural coloration in nature

**Fig. 4:**
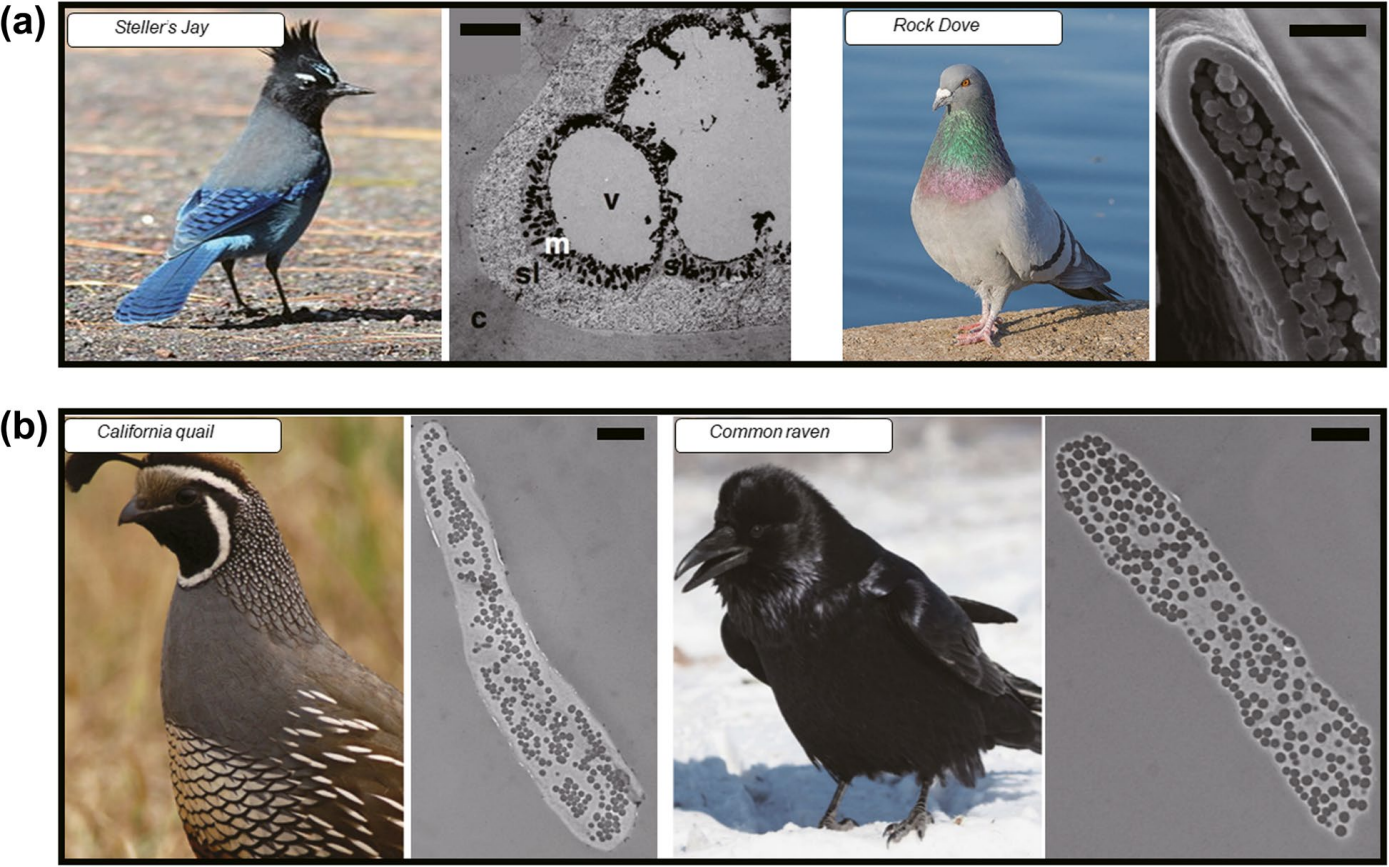
Melanin as an absorbing material of broadband visible wavelengths for coloration. (**a**) Amorphous melanosomes in the blue barb of the Steller’s jay and the barbule of the rock dove’s (domestic pigeon) feathers. (Scale bars: 2 μm). Steller’s Jay, image reproduced under CC BY license. Photograph by Noel Reynolds. Feather microstructure of a blue Steller’s Jay feather, image reproduced with permission (Shawkey and Hill 2006). Copyright 2006, The Company of Biologists Ltd. Rock Dove, image reproduced under CC BY license. Photograph by Diego Delso. Feather microstructure of a Rock Dove feather, image reproduced under CC BY license (Yoshioka et al. 2007). Copyright 2007, The Authors. Published by The Physical Society of Japan. (**b**) Matte black color from the barbule of the California quail’s feather and glossy black color from the barbule of the common raven. (Scale bars: 2 μm). Reproduced with permission (Maia et al. 2011). Copyright 2010, The Royal Society

Melanin is the most ubiquitous pigment in that its ability for broadband absorption of UV to visible light can generate pigmentary colors and patterns in many organisms. In addition to pigment-based coloration, melanin can produce bright and vivid structural colors if melanosomes are arranged into uniform nanostructures. The colors produced here are iridescent, meaning that they change according to the viewing angle. Conversely, non-iridescent colors, colors that do not change with the viewing angle, are often observed in structural colors produced by keratin, not melanin. Melanin acts as an absorbing material of broadband visible wavelengths for coloration. For example, amorphous melanosomes in the blue barb in Steller’s jay indirectly affect non-iridescent colors as shown in Fig. 4(a) (Shawkey and Hill 2006). Amorphous melanosomes in the barb can absorb incoherent scattering from β-keratin spongy layers and improve the color saturation. The barbule of the rock dove (domestic pigeon) is an example of thin-film interference-based structural coloration because the keratin cortex that is on top of the melanosomes is thick enough to produce constructive interference from the air/keratin cortex and keratin/melanosome interfaces (Yoshioka et al. 2007). Melanosomes in the barbule of rock dove provide enough refractive index contrast (melanin ~ 1.8 and keratin 1.54) to produce thin-film interference. Melanosomes also absorb broadband visible light other than constructive interference and contribute to color as a dark background. R. Maia et al. found that the keratin cortex’s thickness also affects the glossiness of feathers (Maia et al. 2011). In their study of a comparison between California quail and common raven feathers indicated that the glossiness decreased as the keratin cortex’s thickness, the melanin layer’s discontinuity, and the number of gaps in the melanin layer increased.

Melanin not only acts as an absorbing material of broadband visible wavelengths for coloration, but melanin granules also produce iridescent structural colors when they are arranged with high crystallinity. Highly crystalline melanosomes produce constructively scattered light, and the wavelengths depend on structural parameters of melanosome arrays. Materials (keratin or air) surrounding melanosomes also affect the structural colors because the effective refractive index changes with keratin and air having different refractive indices (Kinoshita 2008). Melanosomes have solid/hollow shapes, including spherical, rod, and platelet shapes, and their arrangements ranging from a single layer to multilayers (Maia et al. 2013).

**Fig. 5:**
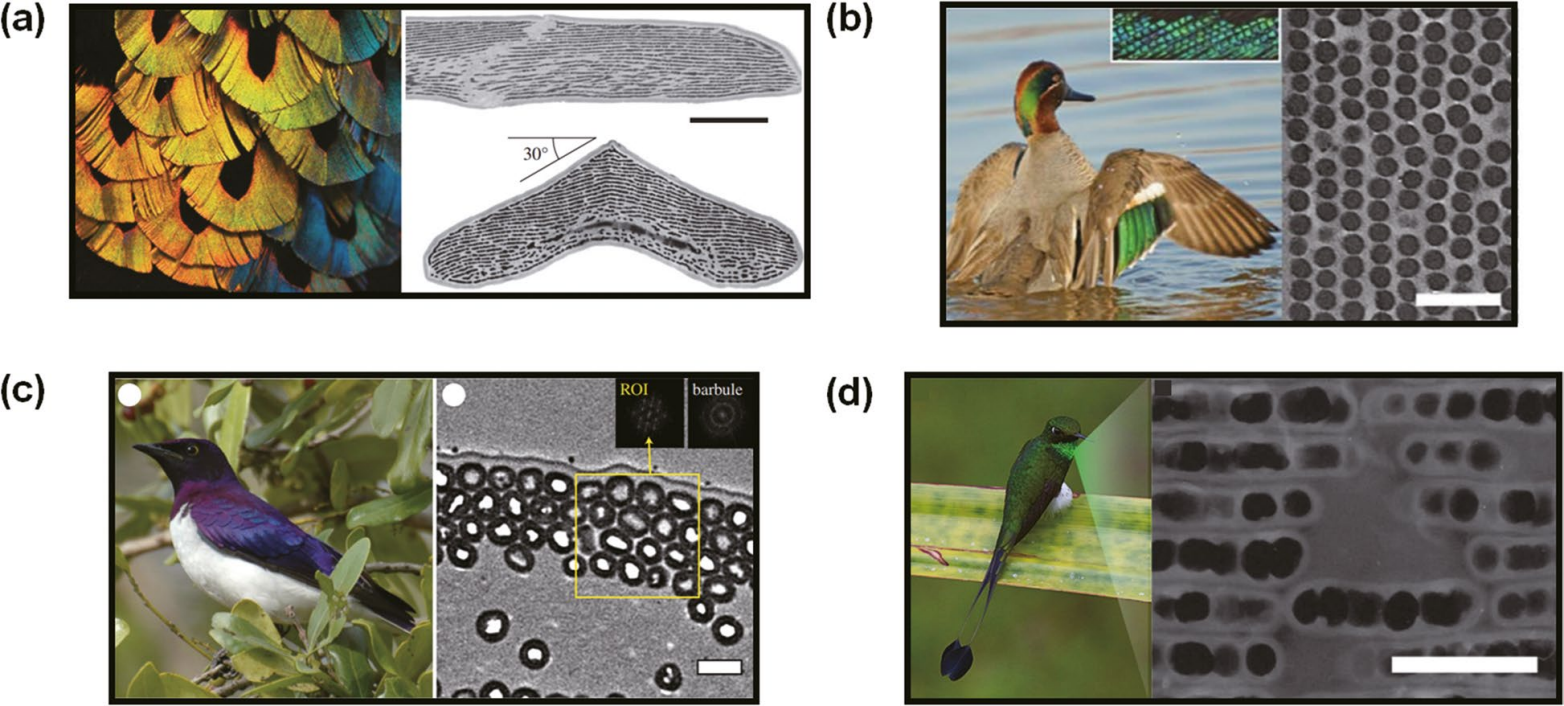
Melanin as a main structural component of structural coloration. (**a**) Multilayer thin films composed of melanosomes in the male Lawes’ parotia’s breast feathers. (Scale bar: 5 μm). Reproduced with permission (Stavenga et al. 2011). Copyright 2011, The Royal Society. (**b**) Non-close packed melanosomes in the wing feathers of the green-winged teal. (Scale bar: 500 nm). Reproduced under the terms of CC BY license (Xiao et al. 2017). Copyright 2017, The Authors. Published by The American Association for the Advancement of Science. (**c**) Close packed hollow melanosomes in the feather barbules of the violet-backed starling. (Scale bar: 500 nm). Reproduced with permission (Eliason et al. 2013). Copyright 2013, The Authors. Published by The Royal Society. (**d**) Layered hollow platelet-shaped melanosomes in the gorget feathers of the white-booted racket-tail hummingbird. (Scale bar: 500 nm). Reproduced with permission (Eliason et al. 2020). Copyright 2020, Wiley

In melanin-based structural colors, thin film interference by stacked melanosomes and keratin is the most basic coloration model (Kinoshita et al. 2008; Kolle et al. 2010; Sun et al. 2013). In birds, established findings demonstrate that multilayer thin film interference can occur due to layers of keratin and melanosomes, which produce iridescent colors (Durrer, and Villiger, W. J. Z. f. Z. u. m. A. 1970; Greenewalt et al. 1960; Hill and McGraw 2006; Land 1972; Zi et al. 2003). Among the basic multilayer thin films composed of melanosomes, one fascinating example is the male Lawes’ parotia’s breast feathers as shown in Fig. 5(a) (Stavenga et al. 2011). They have angular-dependent spectral shifts of reflected light ranging from yellow to blue. These unique optical properties are derived from stacked rod-shaped melanosomes surrounded by keratin layers, and the melanosomes are arranged along boomerang-like barbules.

The most frequently observed melanin-based structural colors are rod-shaped melanosomes arranged along barbules (Li et al. 2010; Liu et al. 2005). Structural parameters that affect the color are mainly classified into four parts: melanosome diameter, pitch (distance between melanosomes), packing density, and orientation. The aspect ratio of eumelanin-dominant melanosome, which in most rod shapes approaches 1:3.7, is also expected to affect color because it affects stacking through depletion attraction of melanosomes (Li et al. 2012; Maia et al. 2012; Piech and Walz 2000). However, the aforementioned factors have a greater impact, thus we do not describe the aspect ratio in this review. Based on the transverse direction of the barbule, melanosomes can arrange from 2D hexagonal close packing to loose packing. Fig. 5(b) represents loosely packed melanosomes in the wing feather of the green-winged teal (Xiao et al. 2017).

Some birds have hollow melanosome structures that increase the intensity of the colors shown on the feathers (Eliason et al. 2013; Maia et al. 2013). The mechanisms for how hollow melanosomes are formed have not yet been determined. However, several studies report that hollow melanosomes could be formed after nanostructural self-assembly of solid melanosomes in developing barbules (D'Alba et al. 2021; Shawkey et al. 2015). This evidence suggests selective loss of the core material in solid melanosomes, possibly due to the disintegration of pheomelanin. Pheomelanin is more soluble in basic solutions, and has less chemical stability than eumelanin (Simon and Peles 2010). Based on this evidence, Shawkey et al. discussed that solid melanosomes with the pheomelanin-core and eumelanin-shell can be the origin of hollow melanosomes and human eyes also contain pheomelanin-eumelanin core-shell melanosomes (D'Alba and Shawkey 2019; Shawkey et al. 2015; Simon and Peles 2010). If we can determine the biological components or conditions that make pheomelanin cores stable in eyes but not in feathers, we will be a step closer to understand the hollow melanosome development process. Eliason et al. suggest that hollow melanosomes allow birds to produce distinct colors compared to solid melanosomes (Eliason et al. 2013), and the increased structural complexity of feather tissues is associated with greater variations in morphology and iridescent coloration (Eliason et al. 2015). They also showed that hollow melanosomes may increase the whole reflectance based on hollow rod-shaped melanosomes of the wild turkey and magpie as shown in Fig. 5(c) (Eliason et al. 2013), and hollow platelet-shaped melanosomes of the hummingbird as shown in Fig. 5(d) (Eliason et al. 2020).

## Biomimetic optical applications using artificial melanin

In recent years, materials for producing structural colors have attracted considerable attention for their use in biomimetic optical applications. Polydopamine (PDA), an artificial melanin, is the most widely used material for artificially implementing structural colors. Similar to natural melanin in birds, artificial melanin’s broadband absorption of UV to visible light helps to increase color saturation of structural colors by suppressing incoherent scattering. Biomimetic structural colors have often displayed low-saturation colors due to the incoherent scattering of light. To suppress the incoherent scattering, absorbing materials such as carbon black have been used conventionally. However, artificial melanin-based structural colors do not need to add extra absorbing materials, because artificial melanin can absorb broadband light and form nanostructures. As PDA can be synthesized in various forms from thin films to core-shell particles, many researchers have used PDA to produce structural colors (Kohri 2019, 2020; Xiao et al. 2020). Self-assembly of colloidal nanoparticles is one of the important and effective methods to produce structural colors. From previous studies, implemented structural colors depend on five parameters: (i) size of nanoparticles (Ge et al. 2014; Kim et al. 2017; Li et al. 2017) and pitch of nanoparticles (Fudouzi and Sawada 2006; Fudouzi and Xia 2003), (ii) refractive index of nanoparticles (Huang et al. 2014; Zulian et al. 2012), (iii) arrangement of nanoparticles (Katagiri et al. 2018; Takeoka 2012; Yoshioka and Takeoka 2014), (iv) absorbance of nanoparticles and substrates (Forster et al. 2010; Takeoka 2018; Takeoka et al. 2013), (v) shape of nanoparticles (Kohri et al. 2019). These five parameters affect the overall optical properties from hue to angle-dependency. Because this review focuses on structural coloration using PDA-based artificial melanin, we will discuss representative examples of how structural parameters (size, arrangement, and shape) affect structural colors.

**Fig. 6:**
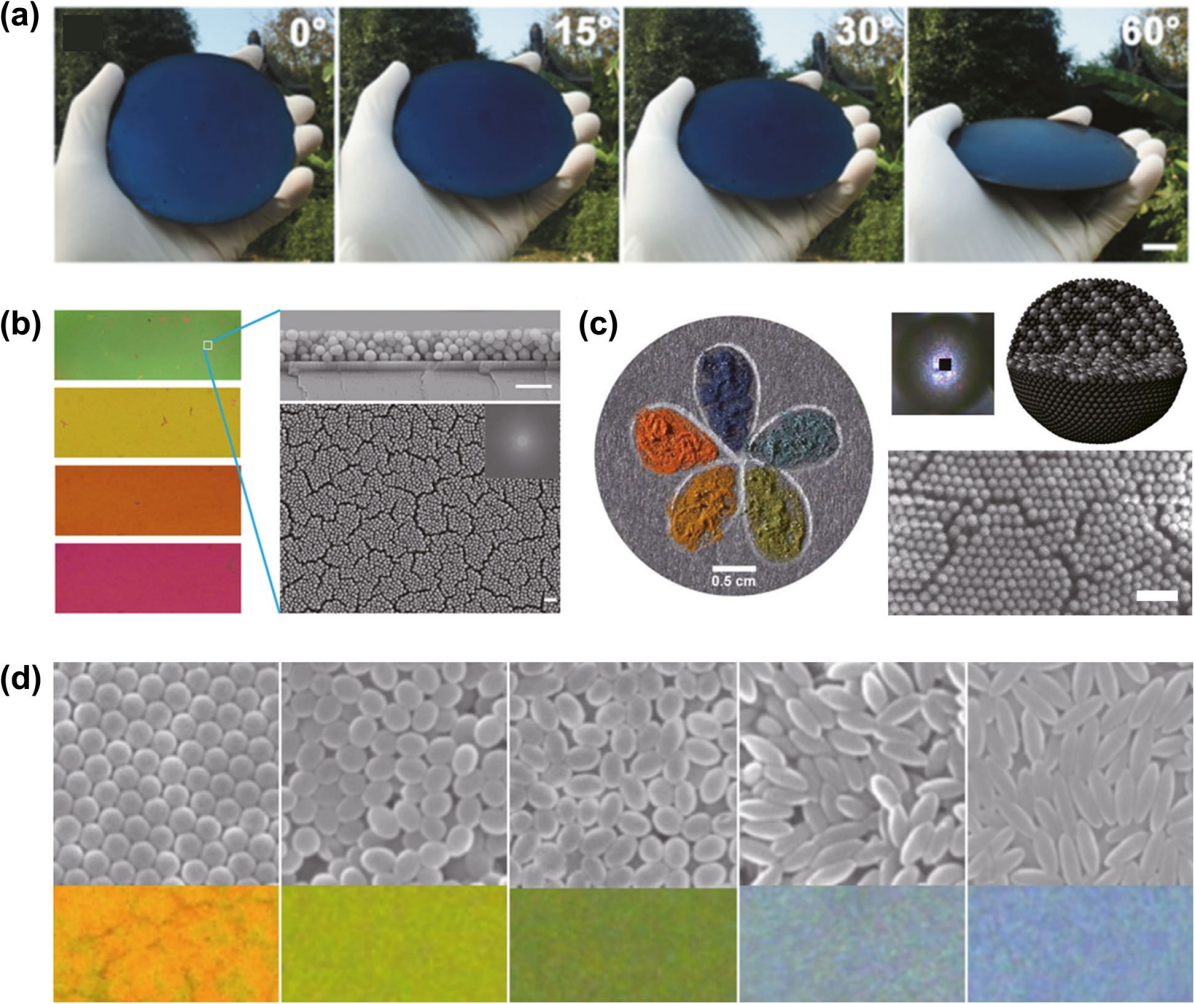
PDA-based artificial melanin for producing structural colors. (**a**) Structural colors using a PDA coating on a silicon wafer. (Scale bar: 1 cm). Reproduced with permission (Zhang et al. 2017). Copyright 2017, Royal Society of Chemistry. (**b**) Structural colors by thin-film interference of PDA nanoparticles. (Scale bar: 500 nm). Reproduced with permission (Xiao et al. 2015). Copyright 2015, American Chemical Society. (**c**) Supraball-type photonic ink from a PDA core and silica shell nanoparticles. (Scale bar: 500 nm). Reproduced under the terms of CC BY license (Xiao et al. 2017). Copyright 2017, The Authors. Published by The American Association for the Advancement of Science. (**d**) Nanoparticles’ aspect ratio dependent structural coloration. Reproduced with permission (Kohri et al. 2019). Copyright 2019, American Chemical Society

As described earlier, PDA has been synthesized in the form of thin films to core-shell particles. The most representative study in thin film-based structural coloration is shown in Fig. 6(a). Zhang et al. reported a simple method that achieves angle-independent structural colors using a PDA thin film coating on a silicon wafer (Zhang et al. 2017). Most of the studies have used spherical-shaped nanoparticles. For instance, Kohri et al. reported that bright structural colors are achieved by core-shell-type artificial melanin nanoparticles (Kohri et al. 2015). In 2015, Xiao et al. reported highly saturated structural colors with thin film structures containing assembled PDA nanoparticles as shown in Fig. 6(b) (Xiao et al. 2015). Xiao et al. also demonstrated full-spectrum non-iridescent colors by supraball ink composed of PDA core and silica shell nanoparticles as shown in Fig. 6(c) (Xiao et al. 2017). Although many researchers have reported bright structural colors using PDA-based artificial melanin, the effect from the shape of nanoparticles is still poorly understood. In nature, rod-shaped anisotropic melanosomes play a significant role for structural coloration, however, there are only a few examples for artificially implementing structural colors using non-spherical nanoparticles. Kohri et al. reported ellipsoidal artificial melanin nanoparticles for structural coloration (Kohri et al. 2019). The anisotropic nanoparticles can be achieved by stretching asymmetrically of polystyrene core and PDA shell spherical-shaped nanoparticles. In addition to color production, biomimetic applications inspired by structural colors in nature have a considerably wide range of applications, ranging from humidity sensor (Xiao et al. 2016) to strain-sensor (Wang et al. 2020), and the range of biomimetic applications is still expanding rapidly. As PDA-based artificial melanin has various functionalities ranging from producing structural colors to UV shields, PDA will play a more significant role in future biomimetic optical applications.

## Conclusion

In this review, we broadly summarized melanin’s structural and optical properties, current understanding of melanin-based structural coloration, and fabrication methods to implement structural colors using synthetic melanin. Information on chemical structures of pheomelanin is less understood than eumelanin. Melanosomes’ shapes vary from spherical, rod, and hollow rod to hollow platelets. Melanosome morphologies may depend on the chemical structures of melanin. We also discussed the optical properties of melanin. Eumelanin and pheomelanin absorb visible light, and eumelanin absorbs UV to blue light more than pheomelanin. Eumelanin and pheomelanin’s absorption spectra do not have peaks. Their monotonic absorption spectra are unusual compared to other biological pigments. Not only melanin acts in pigment as an absorbing material of broadband visible wavelengths for coloration, but melanin granules also produce iridescent structural colors when they are arranged with high crystallinity. We introduced a few studies on the optical advantages of hollow melanosomes, and briefly addressed the need for future research on the biosynthetic mechanisms of hollow melanosomes. Although many optical applications have been implemented based on the understanding of natural melanin granules, continuous research on the natural coloration mechanisms and biological synthetic pathways will be required to open up new possibilities for more energy-efficient biomimetic applications.

## Data Availability

N/A

## Abbreviations

SEM: scanning electron microscopy
TEM: transmission electron microscopy
AFM: atomic force microscopy
EELS: electron energy loss spectroscopy
EDS: energy-dispersive spectroscopy
PhCs: photonic crystals
PhGs: photonic glasses
DHI: 5, 6-dihydroxyl indole
DHICA: 5, 6-dihydroxyindole-2-carboxylic acid
UV: ultraviolet
PDA: polydopamine
